# Kinetics and Mechanism of SiO_2_ Extraction from Acid-Leached Coal Gangue Residue by Alkaline Hydrothermal Treatment

**DOI:** 10.3390/ma17174168

**Published:** 2024-08-23

**Authors:** Deshun Kong, Yuan Gao, Shuojiang Song, Rongli Jiang

**Affiliations:** 1 School of Chemistry and Materials Engineering, Liupanshui Normal University, Liupanshui 553004, China; 2 School of Chemical Engineering and Technology, China University of Mining and Technology, Xuzhou 221016, China

**Keywords:** acid-leached coal gangue residue, alkali leaching, leaching kinetics, silica

## Abstract

Acid-leached gangue residue is produced after the gangue extraction of metal ions; the main component is silicon, which can be used to extract silica. To ascertain the kinetics and mechanism of silica extraction from acid-leached coal gangue residue, this study explored the effects of the NaOH concentration, solid-to-liquid ratio, reaction temperature, and reaction time on the extraction process. The optimized conditions, determined through this investigation, involved a NaOH concentration of 4 mol/L, a reaction time of 4 h, a solid-to-liquid ratio of 1:4, and a reaction temperature of 180 °C, yielding a SiO_2_ extraction ratio of 90.16%. Additionally, the leaching kinetics of silica in a NaOH solution were examined using three kinetic equations from the “unreacted shrinking core model”. The results revealed that the control type of the leaching process was the “mixing control”, and the apparent activation energy was determined to be 52.36 kJ/mol.

## 1. Introduction

Coal gangue is an industrial waste produced during coal mining and washing processes [1,2] and presents environmental challenges. The extraction of aluminum, iron, and other elements from it represents a significant method for resource utilization [3,4,5]. However, the extraction process results in the formation of acid-leached residue, which has the potential to cause secondary pollution [6,7]. The acid-leached residue has a porous structure and contains a small amount of unwashed acid [8]. Improper stacking may result in acid infiltration into the ground through rainwater leaching, polluting the groundwater and eroding the soil, thereby causing irreversible ecological damage [9]. Consequently, the acid-leached residue should be harnessed to protect the environment, extend the coal industry chain, and enhance the industry’s added value [10,11].

Due to the low activity of silicon in coal gangue, a technique based on Fe_2_O_3_–alkali leaching and silica activation roasting was proposed [12]. During the roasting process, the Al–Si bond in the aluminosilicates breaks. The calcined products can be leached with NaOH solution [13,14], and a sodium silicate leaching solution is obtained. However, this method requires the separation of iron and silicon elements, and the operation is complicated.

Furthermore, when SiO_2_ is extracted directly from calcined gangue powder using a NaOH solution, SiO_2_ and Al_2_O_3_ exhibit simultaneous leaching behavior in the alkaline solution [15], while Si cannot be well separated [16]. In addition, sintering in alkaline solutions consumes a lot of energy [17]. In this study, the use of an alkaline solution to extract Si from acid-leached gangue residue is considered, which has the advantages of low energy consumption and a low cost. Metal elements such as aluminum and iron have been dissolved from calcined gangue powder with acid, and Al_2_O_3_ and SiO_2_ have been successfully separated [18].

The primary component of the acid-leached residue is SiO_2_ [18,19,20], comprising quartz and amorphous SiO_2_. Quartz, with its crystal structure, exhibits relative stability and low chemical activity [21,22], whereas amorphous SiO_2_ primarily originates from the decomposition of calcined kaolin, displaying high activity [23,24]. The core objective of silica extraction is to facilitate the reaction of silica within the residue with other substances to generate soluble silicates. The various methods for the extraction of silica include fluoride salt sintering [25], carbonate melting [26], water activation [27], etc. However, these methods face challenges such as excessive energy consumption, intricate operation, and low extraction ratios, posing critical bottlenecks for silica extraction technology. Thus, identifying an alkali or salt that can effectively react with SiO_2_ under appropriate conditions is a crucial yet challenging task for the successful extraction of silica and the efficient utilization of acid-leached coal gangue residue.

To study the dissolution kinetics of the extraction of SiO_2_ from calcined gangue, alkaline substances are mostly used, such as Na_2_SiO_3_ and NaOH solutions, and then the “unreacted shrinking core model” is used for calculation. However, the experimental operation and data processing are complicated [28].

Currently, in the field of silica extraction, the reaction kinetics of ores with NaOH solution are investigated using the “unreacted shrinking core model”. This model has found widespread application in studying the kinetics of NaOH solution reactions with various substances, such as fly ash [29], nickel laterite [30], and coal gangue [31,32]. The “unreacted shrinking core model” has been extensively explored, particularly in the context of leaching silicon elements from ores, contributing to a profound understanding of the leaching kinetics. However, there remains a notable scarcity of research on the kinetics concerning the extraction of silica from acid-leached coal gangue residue.

Considering the aforementioned factors, this study systematically enhanced the process conditions for silica extraction using a NaOH solution from acid-leached coal gangue residue through hydrothermal methods. Subsequently, it identified favorable conditions, achieving a high extraction ratio. Moreover, the investigation delved into the leaching kinetics of silica in the NaOH solution, establishing a kinetic model of its leaching process and exploring the dissolution mechanism. This will help researchers to identify the key factors affecting the extraction of silica from the residue and obtain targeted improvements, so as to determine the optimal process conditions and promote the improvement of the production efficiency.

## 2. Materials and Methods

### 2.1. Materials

The acid-leached residue was obtained by leaching Al^3+^, Fe^3+^, and other metal ions from calcined coal gangue obtained from the Wangjiazhai Coal Mine in Liupanshui City, Guizhou Province, China. NaOH, of analytical purity, was sourced from Tianjin, China, Tianjin Aopusheng Chemical Co., Ltd. KBr, of spectroscopic purity, was purchased from Tianjin, China, McLean Reagent Co. Ltd. Deionized water was self-produced.

### 2.2. Methods

**Figure 1:**
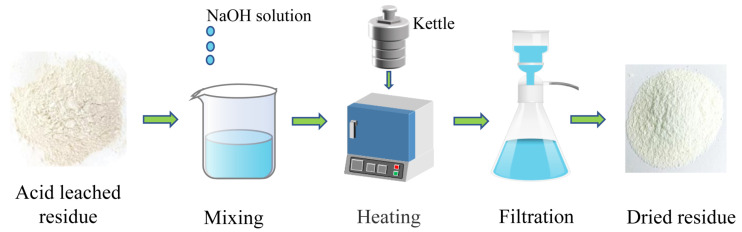
Flow chart of the experiment.

The acid-leached coal gangue residue, with a mass of 10.00 g, as depicted in Figure 1, was combined with a certain concentration of NaOH solution and transferred to a reaction kettle; subsequently, the kettle was placed in an oven at the appropriate temperature. Once the reaction was completed, the mixture underwent filtration, washing, drying, and testing of the mass and the main composition. The extraction ratio of SiO_2_ was then calculated using the following formula [33]:

R = [(mα − m_1_β)/mα] × 100% (1)

where “R” is the SiO_2_ extraction ratio (%); “m” is the mass of the residue before silica extraction (g); “α” is the SiO_2_ content of the residue before silica extraction (%); “m_1_” is the mass of the residue after silica extraction (g); “β” is the SiO_2_ content of the residue after silica extraction (%).

### 2.3. Characterization Techniques

The instruments and working conditions are shown in Table 1.

## 3. Results and Discussion

### 3.1. XRF Analysis of Acid-Leached Coal Residue

The composition of the acid-leached coal gangue residue is presented in Table 2. It shows that the primary components were SiO_2_ and TiO_2_, with residual amounts of Al_2_O_3_, Fe_2_O_3_, and other substances.

### 3.2. XRD Analysis of Acid-Leached Coal Gangue Residue

**Figure 2:**
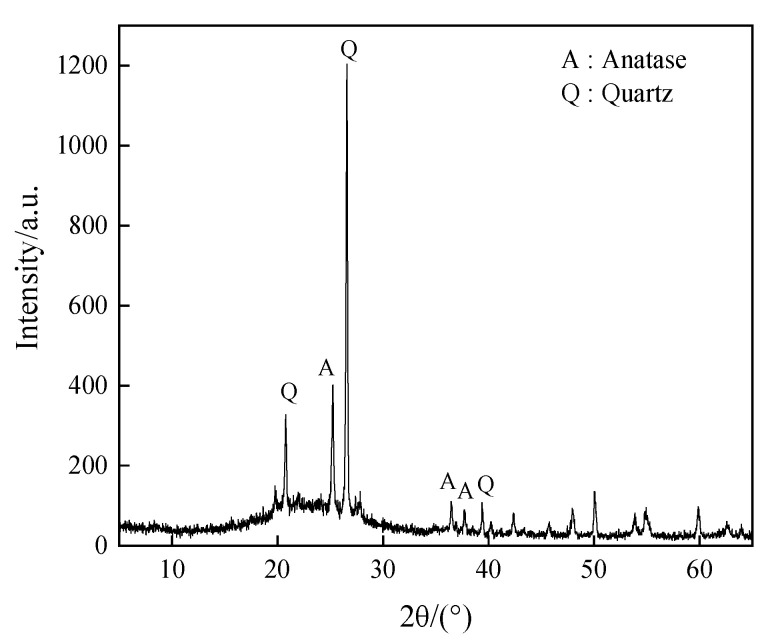
XRD pattern of the acid-leached coal gangue residue.

The XRD analysis of the acid-leached coal gangue residue revealed predominantly crystalline phases, namely quartz (PDF card No. 46–1045) and anatase (PDF card No. 21–1272), as depicted in Figure 2. Complementing the findings from Table 2, the residue most likely contained amorphous SiO_2_, Fe_2_O_3_, Al_2_O_3_, and other compounds lacking distinctive peaks in the XRD spectrum, supporting the results in Table 2.

### 3.3. FT-IR Analysis of Acid-Leached Coal Gangue Residue

**Figure 3:**
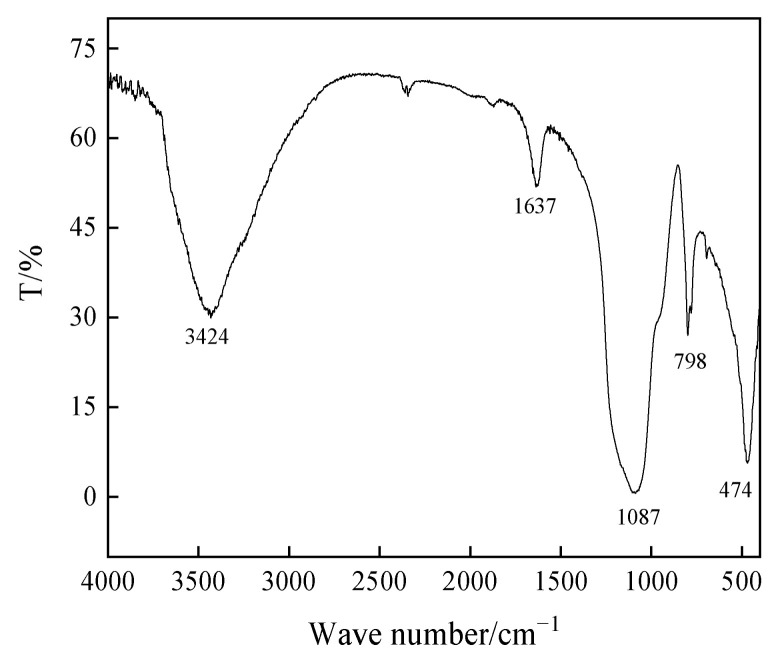
FT-IR spectrum of the acid-leached coal gangue residue.

Notably, the characteristic peak in the range of 3424 cm^−1^ to 3720 cm^−1^ in Figure 3 represents the characteristics of the water molecule adsorption peak [34], with an additional weaker characteristic peak around 1637 cm^−1^ corresponding to the water molecule characteristic peak. The characteristic peak at approximately 474 cm^−1^ confirmed the existence of SiO_2_ [35]. The characteristic absorption bands at about 550 and 440 cm^−1^ can be assigned to the Fe-O vibrations of iron (II) and iron (III) oxides, respectively. In the residue spectrum, the broad and strong characteristic peak near 1087 cm^−1^ was identified as the characteristic peak of SiO_2_ [36]. The broad peak spanning 500 cm^−1^ to 700 cm^−1^ resulted from the stretching vibration of the Ti–O bond [37], and the peak at 798 cm^−1^ was attributed to the Ti–O stretching vibration [38]. This comprehensive analysis affirmed that SiO_2_ and TiO_2_ were the principal components of the acid-leached coal gangue residue, aligning with the conclusions drawn from the XRD and XRF analyses.

### 3.4. Effect of Reaction Conditions on SiO_2_ Extraction

The extraction of SiO_2_ was found to be influenced by key factors, namely the NaOH concentration, solid-to-liquid ratio, reaction temperature, and reaction time, which were systematically investigated through single-factor experiments.

#### 3.4.1. Effect of NaOH Concentration and Solid-to-Liquid Ratio

**Figure 4:**
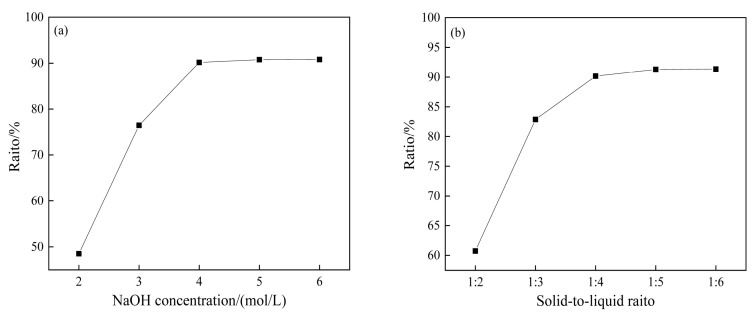
Effect of NaOH concentration (**a**) and solid-to-liquid ratio (**b**) on SiO_2_ extraction ratio.

As shown in Figure 4a, the SiO_2_ extraction ratio exhibited an increasing trend with the rise in the NaOH concentration. At lower concentrations of 2 mol/L and 3 mol/L, the extraction ratio remained lower. However, a significant enhancement was observed when the NaOH concentration reached 4 mol/L, reaching a peak of 90.16%. This phenomenon can be attributed to the higher probability of collision between OH^−^ and the residue at elevated NaOH concentrations, facilitating a more efficient reaction and resulting in a higher SiO_2_ extraction ratio. This behavior is consistent with the reaction [39,40]

SiO_2_ + 2NaOH = Na_2_SiO_3_ + H_2_O (2)

The reaction of SiO_2_ with NaOH to form Na_2_SiO_3_ and H_2_O is relatively stable and easy to carry out when analyzed thermally and kinetically. First of all, the Gibbs free energy change for this reaction is negative, implying that the reaction is able to proceed spontaneously under the given conditions. However, the rate of the reaction is not only dependent on the chemical properties of the reactants but is also influenced by the reaction conditions. Here, the concentration of leaching agent NaOH and the concentrations of NaOH in the diffusion layer and the reaction layer affect the reaction rate.

Further increasing the NaOH concentration led to a marginal improvement in the extraction ratio; therefore, a NaOH concentration of 4 mol/L was deemed optimal.

Figure 4b indicates that a lower solid-to-liquid ratio led to a higher silica extraction ratio. Beyond a solid-to-liquid ratio of 1:4, the extraction ratio did not exhibit significant changes with the increase in the volume of the NaOH solution, indicating that the ratio of 1:4 was more appropriate.

#### 3.4.2. Effect of Reaction Temperature and Time

**Figure 5:**
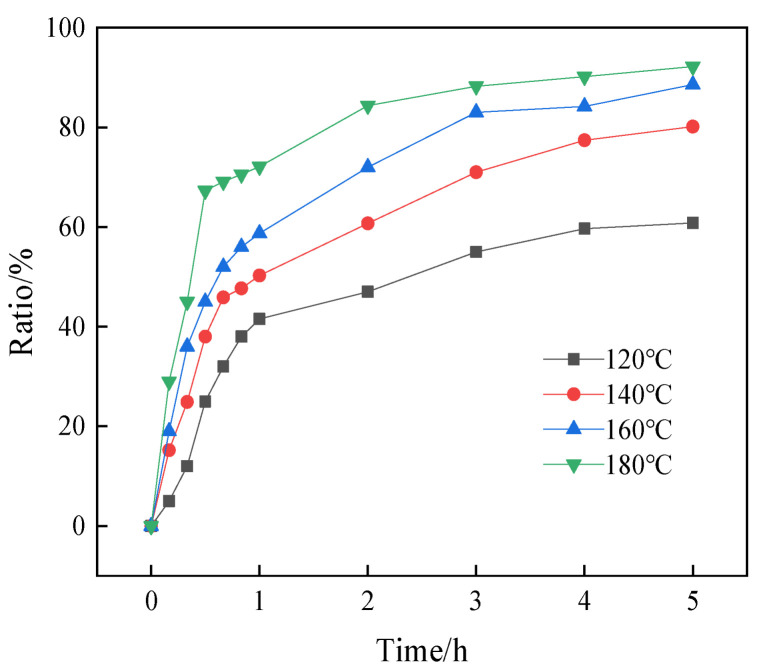
Effect of reaction time and temperature on silica extraction ratio.

In the reaction system with a NaOH concentration of 4 mol/L and a solid-to-liquid ratio of 1:4, reactions were conducted at temperatures ranging from 120 °C to 180 °C, with the extraction ratios determined after 0 to 5 h. As depicted in Figure 5, both the reaction temperature and time were positively correlated with the SiO_2_ extraction ratio; higher temperatures and longer reaction times resulted in increased extraction ratios. Considering the cost-effectiveness, the optimized conditions were determined as follows: a reaction temperature of 180 °C, a reaction time of 4 h, a NaOH concentration of 4 mol/L, and a solid-to-liquid ratio of 1:4.

### 3.5. Kinetics of SiO_2_ Extraction

The study of the dissolution of silicon in the residue is beneficial to obtain the key factors affecting the dissolution rate, which is more effective than the simple optimization of the process conditions. Considering the optimized process conditions, the temperature and time emerged as pivotal factors in the kinetics study. The reaction between the residue and NaOH solution occurred in a typical non-catalytic manner in the liquid–solid phase system. The residue predominantly comprised amorphous silica and quartz crystals, both capable of reacting with the NaOH solution under hydrothermal conditions. However, certain residual components displayed challenging or slow reactions with the NaOH solution, reinforcing the applicability of the “unreacted shrinking core model” [41,42].

In the kinetics analysis of silica extraction, the assumption that “interfacial chemical reaction control” is the governing step leads to the kinetics equation

1 − (1 − x)^1/3^ = k_1_t + b_1_ (3)

If the silica leaching reaction is controlled by “solid film internal diffusion control”, the leaching kinetics equation becomes

1 − (2/3)x − (1 − x)^2/3^ = k_2_t + b_2_ (4)

When the leaching process is simultaneously influenced by “interfacial chemical reaction control” and “solid film internal diffusion control”, denoted as “mixing control”, the kinetics equation takes the form

(1 − x)^−1/3^ − 1 + (1/3)ln(1 − x) = k_3_t + b_3_ (5)

where “k_1_” is the “interfacial chemical reaction control” reaction rate constant, “k_2_” is the “solid film internal diffusion control” reaction rate constant, “k_3_” is the “mixing control” reaction rate constant, “x” is the extraction ratio, “t” is the extraction time, and “b_1_, b_2_, b_3_” are constant terms.

**Figure 6:**
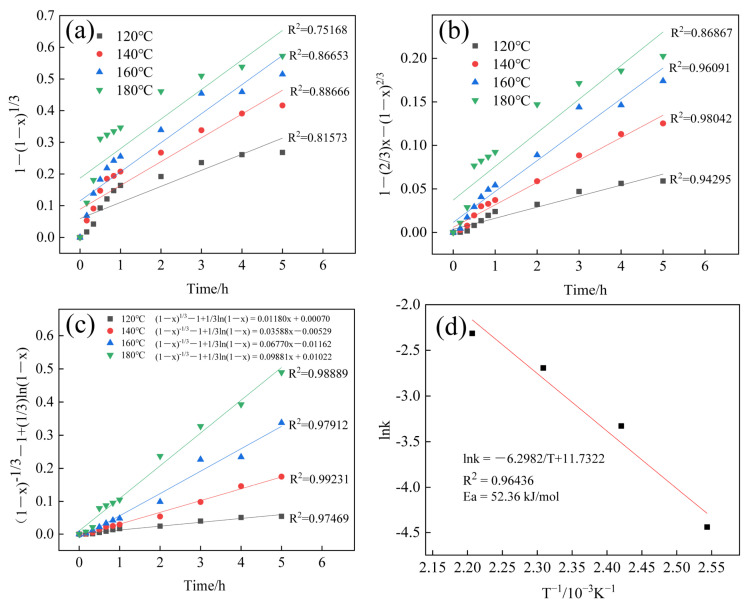
Fitting of silica extraction kinetics data and Arrhenius plot of silica extraction kinetics: (**a**) regression plot of “interfacial chemical reaction control”; (**b**) regression plot of “solid film internal diffusion control”; (**c**) regression plot of “mixing control”; (**d**) Arrhenius plot of silica extraction kinetics.

Figure 5 illustrates the variation in the silica extraction ratio with the temperature and time, and Figure 6 delves into the kinetics of the reaction using the three mentioned kinetic models. The results indicate that the fitted equation of the “mixing control model” (Figure 6c) yielded superior results (closer to R^2^ = 1) compared to the other two models (Figure 6a,b), affirming that the extraction process was controlled by “mixing control”. Furthermore, the apparent activation energy was determined to be 52.36 kJ/mol based on the Arrhenius equation k = Aexp [–E_a_/(RT)], as shown in Figure 6d.

The findings are very helpful in enabling us to adopt reasonable technological conditions to improve the extraction ratio of silica from acid-leached residue.

### 3.6. Reaction Mechanism

#### 3.6.1. XRD Patterns of Acid Residues after Silica Extraction

(1) Effect of NaOH concentration on SiO_2_ extraction

**Figure 7:**
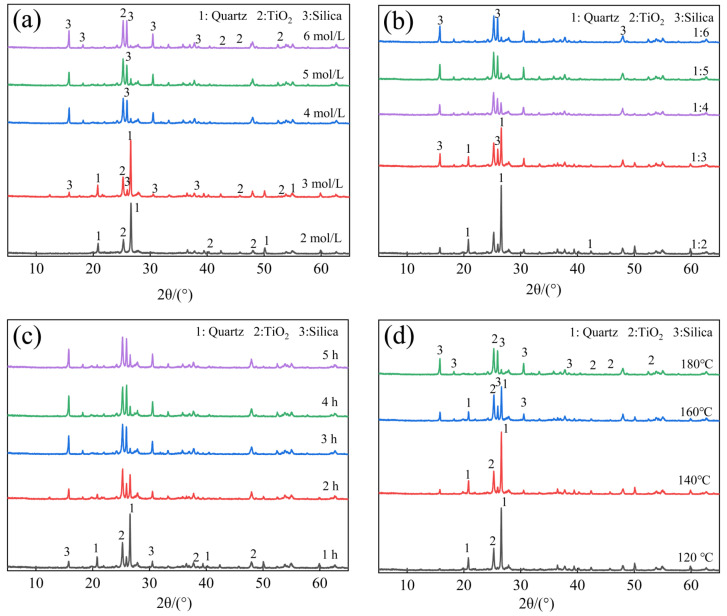
XRD patterns of the residues after silica extraction: (**a**) effect of NaOH concentration; (**b**) effect of solid-to-liquid ratio; (**c**) effect of reaction temperature; (**d**) effect of reaction time.

As seen in Figure 7a, the residue after the extraction of SiO_2_ predominantly comprised SiO_2_ and TiO_2_, with crystalline SiO_2_ primarily in the form of quartz (PDF card No. 46–1045). Characteristic peaks appeared at 2θ = 20.859°, 26.639°, 40.299°, and 50.621°, representing quartz crystals. The anatase (PDF card No. 21–1272) characteristic peaks appeared at 2θ = 25.28, 37.80, and 48.05.

As the NaOH concentration increased, the intensity of the quartz diffraction peaks gradually diminished, eventually nearly disappearing. This phenomenon indicated the progressive dissolution of both the amorphous SiO_2_ and quartz crystals in the NaOH solution under the specified temperature and autogenous pressure conditions [43].

Beyond a NaOH concentration of 3 mol/L, new SiO_2_ crystals (PDF card No. 32–0993) emerged with characteristic peaks at 2θ = 15.478°, 18.785°, 19.981°, and 31.361°. The intensity of these peaks heightened with the increasing alkali concentration, suggesting the re-crystallization of the dissolved silica [44]. This finding emphasizes that excessively high alkali concentrations are not conducive to efficient silica extraction.

(2) Effect of solid-to-liquid ratio on SiO_2_ extraction

Figure 7b illustrates that as the volume of the NaOH solution increased, the intensity of the quartz characteristic peaks continued to weaken, while the diffraction intensity of the newly crystallized SiO_2_ crystals increased. Further increasing the alkali solution’s volume resulted in enhanced diffraction peaks for the fresh SiO_2_ crystals, leading to a reduction in the silica extraction ratio. At a solid-to-liquid ratio of 1:4, the diffraction peaks of the quartz crystals and freshly generated SiO_2_ were lower, indicating a higher silica extraction ratio, consistent with the findings in Figure 4b.

(3) Effect of temperature on SiO_2_ extraction

Figure 7c demonstrates that a higher extraction temperature corresponded to lower diffraction intensity peaks of residual quartz. At low temperatures, the chemical activity of quartz was low, and the reaction rates were slow. Conversely, at high temperatures, the reaction rate accelerated, with the quartz diffraction peaks nearly disappearing at 180℃, indicating the extensive dissolution of SiO_2_.

(4) Effect of reaction time on SiO_2_ extraction

Figure 7d reveals a decrease in the diffraction intensity of quartz crystals with the reaction time, indicating that, in this hydrothermal system, a longer reaction time and higher temperature promoted the dissolution of the amorphous silica and quartz [45,46]. Simultaneously, when the reaction reached a certain extent, a supersaturated solution of sodium silicate was formed in the system. After the supersaturated solution was disturbed, new SiO_2_ crystal nuclei were formed and gradually grew, and there was notable growth in fresh SiO_2_ crystals during the later stages, indicating a concurrent process of silica dissolution and the re-crystallization of new SiO_2_ crystals within the system [47]. At the beginning of the reaction, the silicate concentration in the system was low; as the reaction progressed, the concentration gradually increased. During this phase, the nucleation and growth of fresh SiO_2_ crystals took place. Concurrently, the original quartz crystals continued to dissolve, providing new raw materials. Consequently, the new nuclei continued to acquire the necessary “nutrients” for the growth of fresh SiO_2_ crystals from the system [48,49]. This dynamic interplay increased the diffraction intensity of the fresh SiO_2_ crystal peaks with an extended reaction time.

In the early stage of the reaction, as the reaction between the silica and sodium hydroxide solution proceeds, the concentration of silicate in the solution gradually increases. When the reaction reaches a certain extent, a supersaturated solution of sodium silicate is formed in the system. After the supersaturated solution is disturbed, new SiO_2_ crystal nuclei are formed and gradually grow, which is the process of dissolution–recrystallization.

Therefore, it is beneficial to control the conditions appropriately to obtain a higher extraction rate of silica.

#### 3.6.2. FT-IR Analyses of Silica Extraction Residues

**Figure 8:**
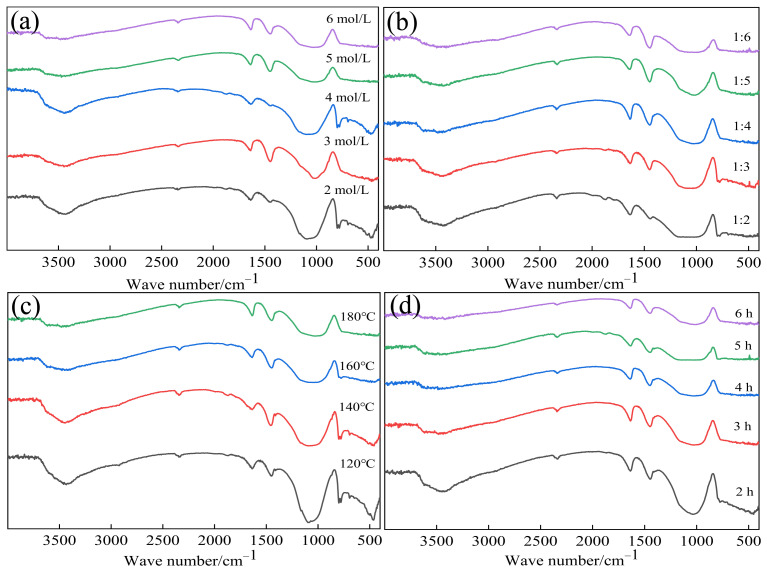
FT-IR spectra of residues after extraction of silica: (**a**) effect of NaOH concentration; (**b**) effect of solid-to-liquid ratio; (**c**) effect of reaction tesmperature; (**d**) effect of reaction time.

The silica extraction residues were obtained as in Section 3.6.1, and the FT-IR spectra of the residues are presented in Figure 8a–d, demonstrating their similarity after silica extraction. In all spectra, a prominent characteristic peak at 1018 cm^−1^ indicated the anti-symmetric telescopic vibrational characteristic peak of Si–O–Si [50]. The characteristic SiO_2_ peaks at 470–798 cm^−1^ overlapped with the distinctive TiO_2_ peaks (500–700 cm^−1^). The increase in the SiO_2_ extraction ratio led to a decrease in the SiO_2_ content in the residue. The superposition of two characteristic peaks resulted in a reduced total characteristic peak intensity, making it challenging to discern an increase in the Ti–O characteristic peak intensity in the spectra.

#### 3.6.3. SEM and EDS Analyses of Residues before and after Reactions

**Figure 9:**
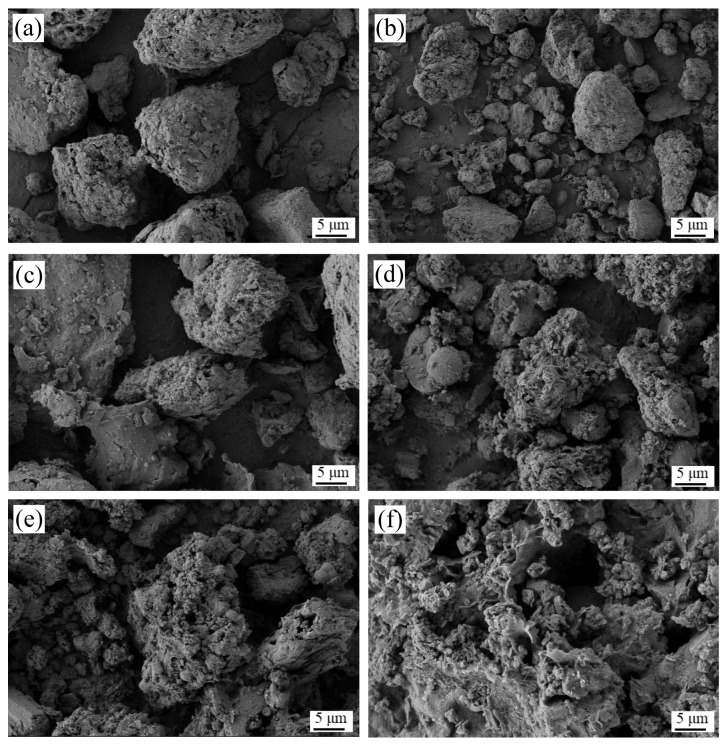
SEM images of the acid-leached coal gangue residue during silica extraction: (**a**) acid-leached residue; (**b**) extraction after 15 min; (**c**) extraction after 30 min; (**d**) extraction after 1 h; (**e**) extraction after 3 h; (**f**) extraction after 5 h.

The morphological changes observed in Figure 9 illustrate the evolving structure of the residue during the silica extraction process. The irregularly spherical particles before extraction became looser and more irregular after 15 min, with a significant transformation after 30 min and further evolution at 1 h, 3 h, and 5 h. These changes suggest the applicability of the “unreacted shrinking core model” to describe the silica extraction process.

**Figure 10:**
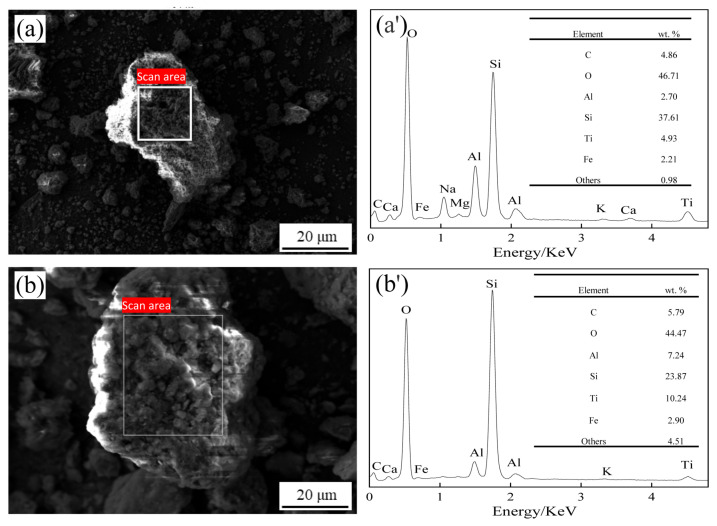
SEM-EDS analyses of the residues before silica extraction (**a**,**a′**) and after silica extraction (**b**,**b′**).

In Figure 10, the SEM-EDS analysis results display alterations in the silicon content and other elements before and after silica extraction. The silicon content decreased from 37.61% to 23.87%, indicating substantial silicon extraction.

#### 3.6.4. Kinetic Modeling Process Analysis

**Figure 11:**
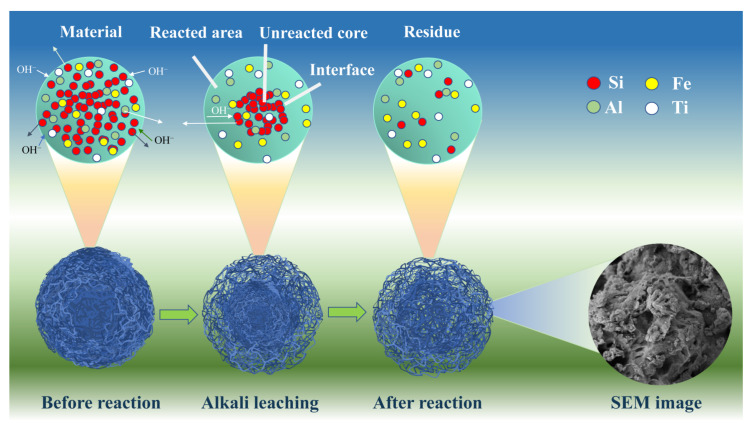
Schematic illustration of SiO_2_ extraction from acid-leached gangue residue.

As discussed above, the primary component of the acid-leached coal gangue residue was SiO_2_, followed by minor amounts of Al_2_O_3_, Fe_2_O_3_, TiO_2_, and other substances. During the alkali dissolution process, NaOH reacted with SiO_2_, leading to the diffusion of the products from the residual solid layer. The reaction interface progressively narrowed towards the core of the residue, and the solid layer thickened, possibly forming flakes. Thus, the “unreacted shrinking core model” aptly describes the silica extraction process. Figure 11 depicts the kinetic process of silica extraction, involving steps such as OH^−^ ions reacting with the particle surface, OH^−^ diffusion into the particle interior, the formation of a reaction interface through OH^−^ ions reacting with silica, the outward diffusion of silicate products through the residual solid layer, and, finally, the diffusion of silicate products into the solution.

Our work faces a challenge or problem to be solved: we must take the necessary measures to promote the chemical reaction according to the key factors affecting the “mixing control”, promote the rapid progress of diffusion, and thus improve the leaching rate of silica. Next, we plan to focus on these two aspects to further improve the leaching rate of silica.

## 4. Conclusions

In this paper, the technological conditions of silica extraction from the acid-leached residue of coal gangue are studied, and the extraction kinetics and mechanism of silica extraction from acid leaching residues are determined. The main conclusions are as follows.

(1) The optimized conditions for silica extraction from the acid-leached residue were a NaOH concentration of 4 mol/L, a solid-to-liquid ratio of 1:4, a reaction temperature of 180 °C, and a reaction time of 4 h, resulting in a SiO_2_ extraction ratio of 90.16%.

(2) The kinetics of SiO_2_ extraction from acid-leached coal gangue residue via a NaOH solution were explored using the “unreacted shrinking core model”. The analysis revealed a dual control mechanism involving “interfacial chemical reaction control” and “solid film internal diffusion control”, indicating a “mixing control” process, which lays a foundation for the development of corresponding measures to improve the dissolution ratio of silica.

(3) The mechanism analysis elucidated that the quartz in the acid-leached gangue residue could effectively react with NaOH under hydrothermal conditions, leading to a higher silica extraction ratio. Furthermore, a higher alkali concentration, increased reaction temperature, and prolonged heating time contributed to the recrystallization of the dissolved SiO_2_, ultimately reducing the silica extraction ratio.

## Figures and Tables

**Table 1:** A list of the main instruments.

Instrument	Type	Production Location	Working Conditions	Usage
X-ray fluorescence spectrometer (XRF)	Supermini 200	Rigaku Corporation, Tokyo, Japan	Power: 4 kW; maximum voltage; and current: 60 kV, 160 mA.	Component analysis
X-ray polycrystalline diffractometer (XRD)	6100	Shimadzu Corporation, Kyoto, Japan	Cu Kα_1_ (λ is Kα = 1.54059 Å); 2θ = 5° (min)–65° (max); step: 0.02°; voltage: 40 kV; current: 35 mA	Phase analysis
Scanning electron microscope (SEM)	EVO18	Analytisches Instrument, Jena, Germany	The surface of the sample is sprayed with carbon and amplified 1000–2000 times	Morphology analyses
Energy-dispersive X-ray spectroscopy (EDS)	Extreme	Oxford Instrument Corporation, Oxford, UK	Map scanning analysis	Energy spectrum analyses
Fourier transform infrared spectrometer (FT-IR)	7600	Tianjin Gangdong Science and Technology Corporation, Tianjin, China	Background calibration with KBr at a ratio of m(sample): m(KBr) = 1:500, and the spectral analysis range was from 4000 cm^−1^ to 400 cm^−1^	Infrared spectral analysis

**Table 2:** Main composition of the acid-leached coal gangue residue (wt.%).

SiO_2_	Al_2_O_3_	Fe_2_O_3_	CaO	TiO_2_	K_2_O	Na_2_O	LOI
78	2.84	1.28	0.02	15.5	0.78	0.02	1.56

## Data Availability

Experimental data are available from the corresponding author upon reasonable request.
